# Suppressive Effects of EGCG on Cervical Cancer

**DOI:** 10.3390/molecules23092334

**Published:** 2018-09-12

**Authors:** Ying-Qi Wang, Jian-Liang Lu, Yue-Rong Liang, Qing-Sheng Li

**Affiliations:** Tea Research Institute, Zhejiang University, # 866 Yuhangtang Road, Hangzhou 310058, China; yqwan@zju.edu.cn (Y.-Q.W.); jllu@zju.edu.cn (J.-L.L.); yrliang@zju.edu.cn (Y.-R.L.)

**Keywords:** *Camellia sinensis*, EGCG, cervical cancer, human papillomavirus (HPV), anticancer

## Abstract

Cervical cancer is the fourth most common gynecological cancer worldwide. Although prophylactic vaccination presents the most effective method for cervical cancer prevention, chemotherapy is still the primary invasive intervention. It is urgent to exploit low-toxic natural anticancer drugs on account of high cytotoxicity and side-effects of conventional agents. As a natural product, (-)-epigallocatechingallate (EGCG) has abilities in anti-proliferation, anti-metastasis and pro-apoptosis of cervical cancer cells. Moreover, EGCG also has pharmaceutical synergistic effects with conventional agents such as cisplatin (CDDP) and bleomycin (BLM). The underlying mechanisms of EGCG suppressive effects on cervical cancer are reviewed in this article. Further research directions and ambiguous results are also discussed.

## 1. Introduction

Cervical cancer is the fourth most common female cancer in terms of both incidence and mortality rates worldwide, with an estimated 528,000 new cases in 2012, including 266,000 deaths (85% occurring in developing countries) [1]. Although screening methods and early detection programs have been established, invasive cervical cancer still represents a major concern for public health. Risk factors of cervical cancer include human papillomavirus (HPV), sexual behavior beginning at a young age (<16 years old), multiple sexual partners (more than four), history of genital warts, HIV positive, and cigarette smoking or environmental tobacco smoke [2]. More than 99% of cervical cancer patients carry at least one genotype of oncogenic HPV [3], since persistent infection with HPV is the prominent etiological reason in the formation of cervical cancer [4]. However, more than 200 types of identified HPVs can be classified as low-risk HPVs and high-risk HPVs [5]. Low-risk HPVs induce inconspicuous infection or benign papilloma which could eventually be resolved by the immune system and rarely cause neoplasia and carcinogenesis [6]. On the contrary, high-risk HPVs are related to the propensity of malignant progression of virus-mediated lesions [7,8]. Among them, HPV16 and HPV18 are the two major viruses responsible for approximately 70% of all cervical carcinomas worldwide. HPV has two vital transcriptional units, E6 and E7, that encoded oncoproteins primarily attribute to its oncogenic function [9]. E6 protein inhibits the activity of tumor suppressor P53, and E7 protein targets other tumor suppressors of the retinoblastoma family [10,11]. A series of human cervical cancer cell lines have been used to study the potential anticancer ability of chemo therapeutic agents, including HPV18-positive HeLa cell lines, HPV16-positive CaSki and SiHa cell lines, etc. Similar to other cancers, cervical cancer harms the human body mainly due to the proliferation and metastasis of cancer cells. Current treatments in curing cancers aim at anti-proliferation, anti-metastasis of cancer cells, and inducing cancer cell apoptosis.

Until now, prophylactic vaccination is the primarily effective prevention strategy for cervical malignancies [12]. Although these vaccines could prevent approximately 90% of cervical carcinoma, the prohibitive price is incubus especially in developing countries [13,14]. Besides prophylactic vaccination, cervical cancer remains curable if detected at early stage, but hard to remedy in metastatic or recurrent carcinoma [5]. Among conventional therapies including surgery, radiotherapy, chemotherapy and immunotherapy [15], chemotherapy is the first option for patients that could effectively promote the apoptosis of cancer cells. Nevertheless, due to its high chemoresistance ability and toxicity on normal cells, more effective methods using less toxic anticancer drugs and novel therapeutic intervention strategies are required nowadays. Polyphenols such as catechins, curcumin and ferulic acid with low side effects are potential safe anticancer strategies for cervical cancer intervention.

Tea is one of the three most widely consumed non-alcohol beverages in the world. The prominent catechins in teas are (-)-epigallocatechingallate (EGCG), (-)-epicatechingallate (ECG), (-)-epigallocatechin (EGC), and (-)-epicatechin (EC) [16]. EGCG accounts for more than 40% of total catechins in green tea [17], and plays a critical role in cancer chemoprevention, diabetes, neurodegenerative diseases, stroke, obesity and other biochemical disorders [18]. The cancer prevention ability of EGCG is widely supported by results from epidemiological, in vivo and in vitro studies [19,20,21,22], especially in breast cancer [23], liver cancer [24], and prostate cancer [25,26]. However, the effects of EGCG on the prevention of cervical cancer are still inconclusive and controversial [27]. This review summarizes recent research data mainly focused on the effects of EGCG on cervical cancer, including in vivo and in vitro studies, and offers directions for further study.

## 2. Anti-Proliferation of Cervical Cancer Cells

**Figure 1:**
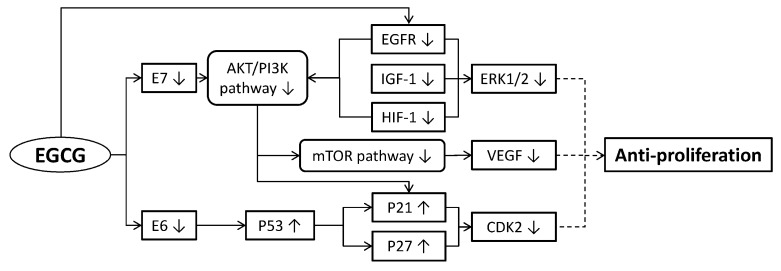
Molecular mechanisms of (-)-epigallocatechingallate (EGCG) suppressing the proliferation of cervical cancer cells. EGCG down-regulated the expressions of E6, E7, EGFR, IGF-1 and HIF-1, then the expressions of downstream targets such as the AKT/PI3K pathway, mTOR pathway and ERK1/2 also declined. The decline of E6 could up-regulated P53 expression resulting in P21 and P27 up-regulations, then CDK2 was down-regulated. The down-regulation of the mTOR pathway led to VEGF reduction. Eventually, the reduced expressions of ERK1/2, VEGF and CDK2 suppressed the proliferation of cervical cancer cells (broken lines mean indirect approaches). AKT: protein kinase B; CDK2: cyclin-dependent kinase 2; E6: one of human papillomavirus (HPV) oncogenes; E7: another HPV oncogene; EGFR: epidermal growth factor receptor; ERK: extracellular signal-regulated kinase; HIF-1: hypoxia-inducible factor 1; IGF-1: insulin-like growth factor 1; mTOR: mammalian target of rapamycin; P21: tumor suppressor protein; P27: tumor suppressor protein; P53: tumor suppressor protein; PI3K: phosphoinositide-3-kinase; VEGF: vascular endothelial growth factor.

The mechanism of tumor progression is based on the proliferation and metastasis of cancer cells [28]. One of the characteristics in advanced malignancies is infinite proliferation of cancer cells. Inhibiting proliferation of cervical cancer cells could stabilize the symptoms of a patient and extend the treatment duration with higher curative potential. EGCG can reduce cervical cancer cell proliferation in various ways (Figure 1), including: (1) inducing cancer cell cycle arrest; (2) regulating cancer cell growth; (3) inducing cellular microtubule depolymerization and inhibiting tubulin assembly; (4) inhibiting angiogenesis; and (5) restraining HPV oncoproteins.

### 2.1. Inducing Cancer Cell Cycle Arrest

Mitosis consists of G1, S, G2 and M phases. EGCG-induced mitosis arrest at low concentration (0–25 μg/mL) in squamous cervical carcinoma Me180 cells [29]. EGCG also could arrest cell division during different phases of the cell cycle in various cell lines. In HeLa cells, EGCG and theaflavins induced sub-G1 phase arrest in a dose-dependent manner and showed a time-dependent inhibition of their proliferations [30]. EGCG induced G1 phase arrested in HPV16-related CaSki cells and regulated related gene expressions [31]. In G2/M phases, EGCG could arrest the cell growth of the HeLa cell line and SiHa cell line in a time-dependent and dose-dependent manner, respectively.

Organisms secrete tumor suppressors to arrest the cell cycle in tumor cells, such as P53 protein and cyclin-dependent kinases inhibitors (CKI) [32]. During cancer cell proliferation, the activation of epidermal growth factor receptor (EGFR) and its downstream target, extracellular signal regulated protein kinases ERK1/2, are also required. By increasing the level of P53 and CKIs (P21/WAF-1, P27/KIP-1), EGCG inactivated EGFR and ERK1/2 protein kinases, resulting in G1 arrest and increasing cell apoptosis in several cervical cancer cell lines finally [33]. Moreover, EGCG increased the expression of P53 dose-dependently. Besides, insulin-like growth factor receptor (IGF-1R) is a tyrosine kinase receptor which could activate the ERK signaling pathway by recruiting the Src homolog-2 domain transforming protein, and has been confirmed to induce mitosis, transformation and anti-apoptosis of cancer cells [34]. EGCG effectively decreased the phosphorylation of ERKs in a dose-dependent manner, meanwhile, reduced activity of IGF-1R and decreased its binding ability with insulin-like growth factor (IGF-1), result in inhibiting the proliferation and anchorage-independent transformation of HeLa cells [35]. In G2 arrest, the potential mechanism was that EGCG upregulated the expression of P21, an inhibitor of cyclin/cyclin-dependent kinase complexes, which induced mitosis arrest [36]. Otherwise, Polo-like kinase 1 (PLK1) plays a key role in mitotic progression and cell-cycle control [37], which is confirmed to be one of the potential drug targets for cancer therapy [38]. By inhibiting the binding of phosphopeptide to C-terminal of polo-box domain (PBD), green tea catechin containing EGCG was found to be the potent inhibitor of PBD in PLK1. In addition, EGC, as another kind of catechins, also could lead cell-cycle arrest in S and G2/M phases by disturbing the proper cellular localization of PLK1, finally inducing apoptosis in several cancer cells including HeLa cells [39].

### 2.2. Regulating Cancer Cell Growth

Current research reveals that EGCG could regulate cancer cell growth through telomerase and RNA polymerase III. Telomerase, as a reverse transcriptase, adds new DNA onto the telomeres that are located at the ends of chromosomes [40]. Recent studies manifested that the telomerase-regulated telomeres’ length stability might correlated with this phenomenon [41]. Thus, inhibiting the expression of telomerase has been considered as an effective method for anticancer protection. EGCG inhibited telomerase activity and more than 90% of the growth rate of both primary human endocervical cells and ectocervical cells [42]. Telomeric repeat amplification protocol assay showed EGCG could also suppress the telomerase activity in OMC-4 and TMCC-1 cervical adenocarcinoma cell lines [43].

RNA polymerase III (RNA pol III) transcribes RNA processing and related RNA molecules, such as U6 snRNA and tRNA, and dictates the growth rate of a cell [44]. It has been proved that the activity of RNA pol III will be deregulated in multiple cancer cells including cervical cancer cells [45]. RNA pol III proper initiation is decided by transcription factor TF III B, while TF III B is initiated from both internal [46] and external [47] RNA pol III promoters. By inhibiting the expression of TF III B subunits Brf1, Brf2 and its promoter, EGCG modulate the RNA pol III transcription and regulate the cell growth of HeLa cell line [48]. Another study showed the combination therapy of EGCG and retinoic acid also seemed to be effective through inducing apoptosis and inhibiting telomerase activity in cervical adenocarcinoma cells [49]. Those results suggest that EGCG may be beneficial in early cervical lesions and cervical adenocarcinoma prevention.

### 2.3. Inducing Cellular Microtubule Depolymerization and Inhibiting Tubulin Assembly

Microtubules are dynamic filamentous cytoskeletal protein structures [50] composed of tubulins including α and β subunits [50]. Some key roles of microtubules are in proliferation, signaling and migration in cancer cells, hence both microtubule and tubulin are crucial therapeutic targets for anticancer drugs. A recent study found that EGCG could inhibit proliferation of HeLa cells through depolymerizing cellular microtubule and restraining tubulin assembly both in cells and cell-free system (IC50 of 39.6 ± 0.63 μM) [36]. EGCG and theaflavins also prevented the reformation of cold-treat cellular microtubule network distortion in HeLa cells [51]. The mechanism of microtubule depolymerization by EGCG is a similar anti-proliferation function to colchicine (a well-known tubulin drug) through bounding to the α subunit of tubulin, and ultimately leading to apoptosis in cervical cancer cells [36].

### 2.4. Inhibiting Angiogenesis

**Figure 2:**
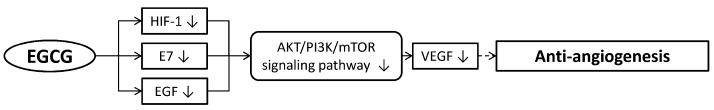
Molecular mechanisms of EGCG inhibiting angiogenesis. EGCG down-regulated the expressions of HIF-1, E7 and EGF, resulting in VEGF down-regulation through AKT/PI3K/mTOR signaling pathways. The decline of VEGF could inhibit angiogenesis (a broken line means indirect approach). AKT: protein kinase B; E7: another HPV oncogene; EGF: epidermal growth factor; HIF-1: hypoxia-inducible factor 1; mTOR: mammalian target of rapamycin; PI3K: phosphoinositide-3-kinase; VEGF: vascular endothelial growth factor.

Angiogenesis consists of vascular endothelial degradation, adhesion and migration procedures, that provide nutrients and oxygen in tumor growth and proliferation. Therefore, inhibiting angiogenesis could effectively suppress the growth and proliferation of tumor cells. Recent work revealed that EGCG could interfere with cell signaling pathways of angiogenesis in ovarian, lung, breast and cervical cancer cells (Figure 2) [52,53,54].

Vascular endothelial growth factor (VEGF) is the key regulator of physiological angiogenesis [55] which can significantly stimulate proliferation and division of vascular endothelial cells, resulting in enhancing the formation of new blood vessels. The expression of VEGF in a tumor is activated by hypoxia-inducible factor 1 (HIF-1) under cellular hypoxia condition [56] and upregulated by protooncogene RAS, SRC, HER2. Hypoxia-inducible factor-1α (HIF-1α) is the subunit of HIF-1 [57], which plays a critical role in tumor growth, angiogenesis [58] and apoptosis [59]. In cervical carcinoma HeLa cells, EGCG and green tea extract effectively inhibited the accumulation of hypoxia-induced HIF-1α protein through blocking PI3K/Akt and ERKs signal pathways, and promoted the degradation of HIF-1α protein via the proteasome system [60]. Moreover, by reducing downstream substrates’ phosphorylation or inhibiting Akt activation directly, EGCG could modulate the activity level of Akt to inhibit epidermal growth factor (EGF)-dependent signaling pathway transportation and restrict HeLa cell proliferation [33]. As a result, EGCG and green tea extract reduced the expression of VEGF at both protein and mRNA levels [61]. Functionally, EGCG and green tea extract abolish the migration of hypoxia-stimulated HeLa cells.

Modulating the expression of the angiogenesis signaling cascade may be one of the potential anticancer mechanisms of EGCG. Transcriptional analysis revealed that EGCG modulated the transcription level of 11 genes in angiogenesis process. Among them, EGCG down-regulated 7 gene expressions including PDGFA (platelet-derived growth factor α, promoting angiogenesis through paracrine mechanism [62,63]), THBS-1 (thrombospondin 1, promoting vascular adhesion, migration and invasion through integrin pathways [64]), CCL2 (monocyte chemoattractant protein 1, related to the recruitment of tumor-infiltrating macrophages [65]), EFNA1 (ephrin A1, a prototype ligand [66]), TGF-β2 (transforming growth factor β 2), TNFAIP2 (tumor necrosis factor α-induced protein 2), and CXCL6 (granulocyte chemotactic protein 2); meanwhile, EGCG up-regulated 4 gene expressions consisting of ANGPTL4 (angiopoietin-like 4, inhibiting vascular invasion [67]), IFN-β1 (interferon β 1, reducing pro-angiogenic factors [68] and blocking endothelial cell migration [69]), IL-1β (interleukin 1 β, decreasing the secretion of matrix metalloproteinase (MMP)-2 in cervical cancer [70]), and ID1 (inhibitor of DNA binding 1). These genes are known as mediating multiple mechanisms in proliferation, adhesion, migration and invasion of vascular endothelial cells [71]. The results suggest that EGCG may act as an important anti-angiogenic agent in cervical cancer.

### 2.5. Restraining Human Papillomavirus (HPV) Oncoproteins

Since persistent infection with HPV is the prominent etiological reason in the formation of cervical cancer [4], seeking drugs for restraining HPV-related oncoproteins is essential. EGCG has a positive effect on suppressing HPV oncogenes and oncoproteins.

E6 and E7 of HPV are two main encoded oncoproteins in cervical cancer. In conjunction with the cellular ubiquitin ligase E6AP, E6 oncoprotein could degrade tumor suppressor protein P53 via the ubiquitin-proteasome pathway; meanwhile, E7 oncoprotein could induce retinoblastoma tumor suppressor gene product pRb degradation [72], resulting in disrupting cell cycle regulation and inhibiting apoptosis of cervical carcinoma [73]. Thus, restraining the expressions of E6 and E7 oncoproteins and their proteasome pathways could inhibit HPV infection and the development of cervical cancer. By accumulating ubiquitinated proteins and natural proteasome targets including tumor suppressor protein P27, IκB-α and Bcl-2 associated X protein (BAX) in HeLa cells [74], EGCG potently inhibited the degradation ability of E7 on pRb through restraining ubiquitin-proteasomal activity and suppressing tumor growth [75,76]. Estrogen also has been confirmed to play a critical role in HPV positive cervical cancer. Aromatase, as the key enzyme in estrogen biosynthesis, could upregulate the expression of estrogen receptor (ER). EGCG could suppress the mRNA and protein expression levels of estrogen receptor-α (ER-α) and aromatase, hence restraining E6 and E7 expressions. As a result, EGCG indirectly inhibits the proliferation and induces the apoptosis of cervical cancer cells [77]. Immunohistochemistry study shows a similar result in both HeLa cells and HPV immortalized cervical epithelial TCL-1 cells [29].

## 3. Anti-Metastasis of Cervical Cancer Cells

The metastasis of cancer cells includes three key steps, adhesion, migration and invasion. EGCG shows an inhibitory effect on the migratory and invasive ability of cervical cancer cells by regulating the activities of proteolytic enzymes, signal pathways, growth factors/receptors and angiogenesis. Moreover, EGCG could inhibit the spreading of HeLa cells through keeping their round shape from changing to multiple filopodia and lamellipodia, which were the characteristics of spreading cells, hence reducing the adherence rate and migration ability of HeLa cells by 40% and 68% after 48 h respectively [71].

Matrix metalloproteinases (MMPs) are related to several proteolytic events [78] and the tissue inhibitor of metalloproteinase-1 (TIMP-1) is confirmed to directly regulate MMPs activity. Both MMPs and TIMP-1 can be used as tumor therapeutic targets in the clinic [79]. By treating HeLa cells with EGCG, the expression of MMP-9 was down-regulated; in an opposite way, the expression of TIMP-1 was significantly increased in a time-dependent manner [80]. Another study also confirmed that EGCG decreased the expressions of both MMP-2 and MMP-9 in HeLa cells and completely abolished their expressions at 50 μg/mL of EGCG, illustrating the inhibition ability of EGCG on MMP-driven migration in cervical cancer cells. A mixture therapy combined with doxycycline, EGCG had a better effect on inhibiting MMP-2 expression in HeLa cells [81]. VEGF, as a crucial regulator for physiological angiogenesis, also plays a key role in cancer cell metastasis. By interfering in hypoxia-mediated activation of PI3K/Akt and ERK1/2 signaling pathways, EGCG and green tea extract down-regulated the mRNA and protein levels of VEGF that results in inhibiting HeLa cell migration [61].

## 4. Pro-Apoptosis of Cervical Cancer Cells

**Figure 3:**
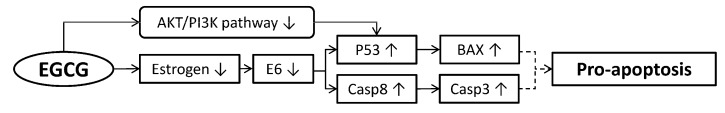
Molecular mechanisms of EGCG promoting apoptosis of cervical cancer cells. EGCG down-regulated E6 through estrogen, then the expressions of P53 and casp8 were up-regulated. EGCG also could increase P53 expression by AKT/PI3K pathways. BAX and casp3 would promote their expressions with up-regulation of P53 and casp8 respectively. The up-regulation of BAX and casp3 could promote apoptosis of cervical cancer cells in the end (broken lines mean indirect approaches). AKT: protein kinase B; BAX: Bcl-2 associated X protein; Casp3: caspase 3; Casp8: caspase 8; E6: one of HPV oncogenes; P53: tumor suppressor protein; PI3K: phosphoinositide-3-kinase.

Anti-proliferation and anti-metastasis of cancer cells stand for restraining malignancies, while pro-apoptosis of cancer cells could cure cancer eventually. EGCG can induce apoptosis of cervical cancer cells (Figure 3) through (1) inducing caspase secretion, (2) ROS induced apoptosis of cancer cells, and (3) inducing lysosomal proteases secretion.

### 4.1. Inducing Caspase Secretion

Caspases are a series of proteases which have been verified as involved in tumor cell apoptosis, necrosis and inflammation. The deficiency of caspase would lead to tumor proliferation. EGCG induced cancer cells apoptosis at high concentration (25–50 μg/mL) in squamous cervical carcinoma Me180 cells [29]. In the meantime, EGCG improved the expression of caspase-3 in cervical carcinoma cells [82]. Black tea polyphenol theaflavins and green tea catechins hydrate could also induce the expressions of caspase-3, -8, -9 and P53 in HeLa [30] or SiHa cells [83]. Eventually, EGCG could promote caspase-mediated cancer cell apoptosis.

### 4.2. Reactive Oxygen Species (ROS) Induced Apoptosis of Cancer Cells

Reactive oxygen species (ROS) are a series of chemical-reactive molecules, containing hydrogen peroxide, superoxide anion radical, singlet oxygen, and hydroxyl radical, which are associated with the decreased antioxidant capability of cells and multiple stages of carcinogenesis [84]. In cancer cells, a low ROS level supports survival pathways that makes a positive impact in cell proliferation, but a medium or high level of ROS causes oxidative stress that could lead to cell apoptosis, necrosis and genotoxic damage [85].

EGCG is not only a natural antioxidant agent in normal cells but also exhibits prooxidant and apoptosis-inducing properties in cancer cells [86,87]. High concentrations of EGCG (50 μg to 200 μg gallic acid equivalents GAE/mL) induced formation of intracellular superoxide anion radical, H_2_O_2_ and hydroxyl radical, as a consequence of increasing oxidative stress in HeLa cells [88]. High-concentration (100 μg gallic acid equivalents GAE/mL) green tea induced the formation of intracellular ROS and inhibited the activity of catalase, while increasing the production of superoxide anion radicals and reducing glutathione. Thioredoxin (Trx) and thioredoxin reductase (TrxR) presenting in all living cells act as antioxidant and apoptotic resistance proteins, which are often overexpressed in drug-resistant cancer cells. Both of them are critical regulators of cellular redox homeostasis. The increased level of ROS induced by EGCG has proven to be an effective regulator for both Trx and TrxR [89]. The inactivation of Trx/TrxR by a high concentration of EGCG was linked to elevation of ROS levels in HeLa cells. By autoxidation, EGCG oxidized into EGCG (semi) quinones and released H_2_O_2_, then inactivated Trx/TrxR through binding with them separately and formed irreversible EGCG-Trx and EGCG-TrxR conjugates, thus inducing prooxidant cytotoxicity and apoptosis of HeLa cells.

Mitochondria, which play an important role in energy production, are major sites of ROS generation [90]. Excessive generation of ROS could lead to the opening of a mitochondrial permeability transition pore with decline in mitochondrial membrane potential (ΔΨm) and consequent release of cytochrome-C from the inter-membrane space into the cytosol, culminating in activation of the caspase cascade and apoptotic cell death pathways [91]. Treating SiHa cells with green tea polyphenols consisting of EGCG decreased ΔΨm of mitochondria and disrupted mitochondrial function, finally inducing apoptosis of SiHa cells [92]. The mitochondrial perturbation ability of EGCG and tea polyphenols may because they can induce the excess hydrogen peroxide.

### 4.3. Inducing Lysosomal Proteases Secretion

Lysosomes are cytoplasmic membrane-enclosed organelles containing hydrolytic enzymes that control the intracellular turnover and degradation of macromolecules [93]. Under lysosomal membrane permeabilization (LMP), lysosomal proteases leak into the cytosol and induce cell death. EGCG was able to trigger LMP supported by Lyso-Tracker Red staining, cathepsin-D cytosolic translocation and cytosolic acidulation, and then lysosomal proteases were released into cytosol and inducing death of HeLa cells [94]. ROS is known to be the principle inducer of LMP. In cancer cell lines, 60 μM EGCG led to intracellular ROS formation which resulted in LMP onset and cytosolic acidification, eventually promoting cell death [94].

## 5. Pharmaceutical Synergistic Effect

Chemotherapy is the most important cancer treatment. But the side-effects of certain chemotherapeutic agents make the human body unbearable before they cure cancers. Chemotherapeutic agents with low side-effects are urgently needed, while seeking chemical compounds with pharmaceutical synergistic effect to reduce the side-effects of chemotherapeutic agents is another effective approach, such as EGCG. Besides, EGCG also has the ability to inhibit cancer cell proliferation, metastasis, and promoting their apoptosis (Table 1).

Cisplatin (cisdiamminedichloroplatinum II, CDDP) is one of the traditional chemotherapeutic agents in the treatment of several types of cancer [95]. The clinical use of CDDP is always limited to its undesirable side-effects, such as nephrotoxicity, gastrointestinal toxicity, neurotoxicity, bone marrow toxicity and ototoxicity [96]. Oxidative stress induced by the strong electrophilic nature of activated CDDP is another common adverse effect of CDDP [97]. Additionally, CDDP-based chemotherapy with concurrent radiation therapy is the commonly used strategy in cervical carcinoma, but the result is unsatisfactory due to its chemoresistance. Therefore, novel combination strategies are required. EGCG has synergistic anticancer activity with CDDP. EGCG could enhance the efficacy of CDDP in inhibiting proliferation of HeLa cells than CDDP alone [98]. Using EGCG combined with CDDP to treat HeLa and SiHa cell lines exhibited significant inhibition of cell growth through blocking kappa-Bα inhibitor phosphorylation, resulting in inhibiting the activation of Akt and NF-κB signaling pathways [99]. Meanwhile, encapsulated polyphenols consisting of EGCG exhibit a higher effect in enhancing the sensitivity of CDDP [100]. TPP@Pt, a nanoparticle synthesized by tea polyphenols with EGCG and platinum, was tested with ability of inducing cell cycle arrest in G2/M phase and increased cell quantity in subG0 death phase in SiHa cells [101]. These findings highlight the synergistic anticancer activity of tea components with CDDP.

Bleomycin (BLM) is another anti-neoplastic chemotherapeutic agent which made by *Streptomyces verticillus*. By inducing DNA oxidative damage and inhibiting cancer cell proliferation [102], BLM is used in redox-related cancer, including testicular cancer and cervical squamous cell cancer [103]. However, BLM also causes several side effects in normal cells, such as immune system damage, hyperpigmentation, pneumonitis and pulmonary fibrosis, which are mediated by redox status disturbances [104,105]. The combination of tea polyphenols with EGCG and BLM could overcome the side effects of BLM and have therapeutic benefits for uterine cervical cancer patients. The combination therapy induced stronger cancer cell apoptosis ability than treated either tea polyphenols or BLM alone, by activating caspase-3, -8, -9 and up-regulating the expressions of P53 and Bcl-2 [106].

## 6. Ambiguous Potential Functions of (-)-Epigallocatechingallate (EGCG) on Cervical Cancer

DNA methylation and histone deacetylase are important epigenetic mechanisms for the inactivation of many genes related to tumor suppressors. Epigenetic alterations are mainly mediated by DNA methyltransferases (DNMT), histone deacetylase (HDAC), and other classes of enzymes [107]. Through targeting epigenetic alterations consisting of DNA methylation, EGCG acts as an epigenetic modifier in bounding the process of carcinogenesis in tumors [108,109]. EGCG significantly reduced the enzymatic activity of DNMT and HDAC in a time-dependent manner in HeLa cells; furthermore, molecular modeling data also supported the result. It was interesting that the expression of DNMT3B normally increased in various cancer tissues and cell lines [110], was decreased in EGCG-treated HeLa cells, whereas there were no significant changes in the expression of HDAC1 [108]. Thus, further studies are needed to determine the efficacy of EGCG for therapeutic purposes as an epigenetic drug.

The 67KD laminin receptor (67LR) is a non-integrin cell surface receptor for extracellular matrix with higher expressions in tumor cells [111]. It is widely related to several processes of cancer cells metastasis [112]. As a major surface receptor of EGCG [113], 67LR could coordinate with EGCG in regulation of several signal pathways and inhibition of tumor growth and promotion of apoptosis. Through 67LR mediation, EGCG inhibited HeLa cells growth by inducing myosin regulatory light chain dephosphorylation [114]. Both eukaryotic translation elongation factor-1A and myosin phosphatase-targeting subunit-1 were considered to be the mediators for EGCG-induced cancer prevention through 67LR in HeLa cells [115]. The sensitivity of 67LR to EGCG would be influenced by the tumor oxidative conditions, high O_2_ pressure suppressed the degradation of ubiquitin/proteasome-mediated 67LR and enhanced the sensitivity of cancer cells [116].

## 7. Further Suggestions

**Figure 4:**
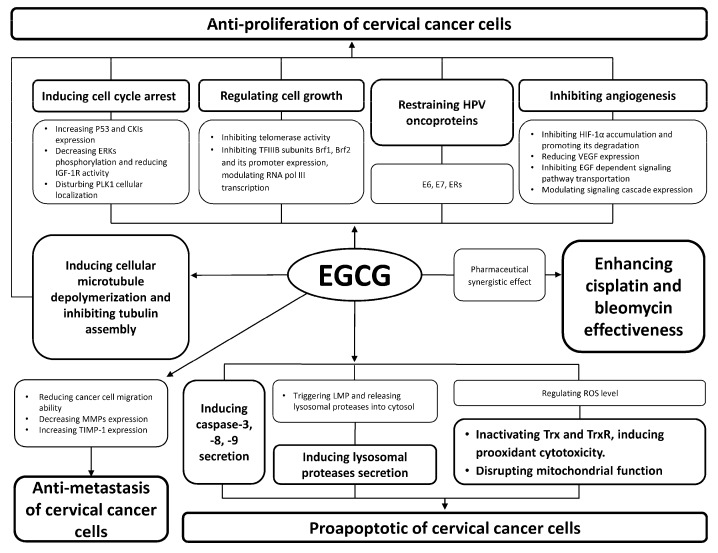
Overview of suppressive effects of EGCG on cervical cancer.

Based on its anti-proliferation, anti-metastasis, pro-apoptosis and pharmaceutical synergistic effects, EGCG shows suppressive effects on cervical cancer. An overview of suppressive effects of EGCG on cervical cancer is illustrated in Figure 4. Meanwhile, the cytotoxic activities of EGCG on human cervical cancer cell lines are summarized in Table 2.

Among anti-proliferation of cervical cancer cells, EGCG depolymerizes the cellular microtubule and binds on tubulin identical to colchicine. However, the IC50 of EGCG is 1000-fold higher than colchicine. This indicates that EGCG may not only target on tubulin of microtubule but also other targets in cellular or extracellular regions [36]. Therefore, further studies should focus on the mechanism of EGCG on depolymerizing cellular microtubule. In addition, EGCG can inhibit telomerase in breast cancer [117], esophageal carcinoma [118], endocervical cells and ectocervical cells [42], but shows much milder cytotoxic effects in cervical cancer cells [119]. The activity of telomerase is related to DNA methylation [120], and thus whether EGCG can inhibit telomerase activity through reducing DNA methylation is a promising research project.

EGCG can induce non-apoptotic cell death through LMP in cervical cancer cells. But the potential mechanism of cervical cancer cell death through upregulating ROS formation has not been certified in HeLa cells [88]. There is only one study focused on the potency of tea components in lysosome-associated cell death pathways [121]. Further studies could work on the mechanism of LMP induced by EGCG in cervical cancer cells.

DNMT and HDAC are two promising targets for anticancer drugs. A series of effective epigenetic targeting drugs in clinical trials has been proved, but adverse reactions such as marrow suppression and gastrointestinal symptoms also appeared during treatment [122]. Thus, a low toxicity natural inhibitor for epigenetic modification is an attractive research direction. EGCG as a dietary agent in cancer treatment has the ability to inhibit the enzymatic activity of DNMT and HDAC in HeLa cells. However, the inhibition mechanism needs more research to support and the efficacy of EGCG for therapeutic purpose as an epigenetic drug should be tested in the future [108].

Although chemoprevention effects and molecular mechanisms of EGCG have been illustrated in cervical cancer, there is only one piece of epidemiological evidence and only one clinical study has been undertaken until now. A case control study in central China showed that green tea intake was identified as a protective factor against cervical cancer or cervical intraepithelial neoplasia [123]. Compared with other cancers [18,19,20], epidemiological research on the suppressive effects of EGCG in cervical cancer is extremely rare.

## 8. Conclusions

EGCG has abilities in the anti-proliferation, anti-metastasis and pro-apoptosis of cervical cancer cells. Although little epidemiological and clinical research supports the suppressive effects of EGCG on cervical cancer, molecular evidence shows positive results of EGCG in inhibiting cervical cancer. Moreover, EGCG could reduce the side-effects of traditional chemotherapy agents, such as CDDP and BLM. Knowledge of more precise and deeper mechanisms of how EGCG restrains cervical cancer cells is required. Overall, EGCG shows a potential role in suppressing cervical cancer.

## Figures and Tables

**Table 1:** Pharmaceutical synergistic effects of EGCG and tea polyphenols.

Ingredient	Drug	Cell Line	Cytotoxic Action	Reference
EGCG	cisplatin	HeLa	Attenuated the toxicity and enhanced the sensitivity of cisplatin, decreased cellular survival and induced apoptosis, regulated NF-kB p65, Akt and mTOR pathways	Kilic et al. (2014) Singh et al. (2011)
	retinoic acid	HEN	Prevented carcinogenesis and induced apoptosis, inhibited telomerase activity	Yokoyama et al. (2008)
Tea polyphenols	platinum	SiHa	Induced G2/M phase cell cycle arrest, increased subG0 cell death phase and inhibited proliferation	Alshatwi et al. (2015)
	bleomycin hydrochloride	SiHa	Enhanced the therapeutic properties of bleomycin (BLM), activated caspase-3, -8, -9, upregulated Bcl-2 and P53 expression and induced apoptosis	Alshatwi et al. (2016)
	cisplatin	HeLa, SiHa	Increased the chemosensitivity and minimized the toxicity of cisplatin	Singh et al. (2013)

**Table 2:** Cytotoxic activities of EGCG on human cervical cancer cell lines.

Cell Line	Function	Cytotoxic Action	Reference
HeLa	Anti-proliferation	Induced G1 phase cell cycle arrest and apoptosis, inhibited EGFR signaling pathway	Sah et al. (2004)
		Induced G2/M phase cell cycle arrest and apoptosis, depolymerized microtubule	Chakrabarty et al. (2011)
		Reduced IGF-1R activity and inhibited proliferation of cells	Li et al. (2007)
		Inhibited Akt and NF-kB activation, inhibited cell growth	Singh et al. (2011)
		Inhibited the expression of Brf1, Brf2 and its promoter, inhibited RNA polIII transcription	Jacob et al. (2007)
		Depolymerized cellular microtubule	Chakrabarty et al. (2015)
		Inhibited HIF-1α protein accumulation, decreased VEGF expression, blocked P3K/Akt, ERK1/2 signaling pathway	Zhang et al. (2006)
		Inhibited HPV E6, E7, ERα, and aromatase expression	Qiao et al. (2009)
		Inhibited proteasome functionality, induced apoptosis	Bonfili et al. (2011)
		Reduced enzymatic activity of DNMT and HDAC, inhibited carcinogenesis	Khan et al. (2015)
	Anti-metastasis	Inhibited invasion and migration, decreased MMP-9 and TIMP-1 expression	Sharma et al. (2012)
		Reduced proliferation, adhesion, invasion of tumor cell, exhibited anti-angiogenesis effect	Tudoran et al. (2012)
		Inhibited invasion and migration, decreased MMP-2, -9 expression	Roomi et al. (2010)
	Pro-apoptosis	Inactivated Trx/TrxR, induced prooxidant cytotoxicity and apoptosis	Zhang et al. (2010)
		Induced LMP secretion	Zhang et al. (2012)
		Induced formation of intracellular ROS	Krstic et al. (2015)
Caski	Anti-proliferation	Induced G1 phase cell cycle arrest and apoptosis, regulated gene expression	Ahn et al. (2003)
		Induced G1 phase cell cycle arrest and apoptosis, inhibited EGFR signaling pathway	Sah et al. (2004)
		Inhibited HPV E6/7, ERα, and aromatase expression	Qiao et al. (2009)
SiHa	Anti-proliferation	Induced G1 phase cell cycle arrest and apoptosis, inhibited EGFR signaling pathway	Sah et al. (2004)
	Pro-apoptosis	Increased caspase-3, -8, -9 secretion and inhibited cell growth	Al-Hazzani et al. (2011)
OMC-1	Anti-proliferation	Inhibited telomerase activity, induced cell cycle dysregulation and apoptosis	Noguchi et al. 2006)
HEN, HEC	Anti-proliferation	Inhibited telomerase activity and cell growth, induced apoptosis	Yokoyama et al. (2004)

## Abbreviations

**AKT:** protein kinase B; **ANGPTL4:** angiopoietin-like 4; **BAX:** Bcl-2 associated X protein; **BLM:** bleomycin; **Casp3:** caspase 3; **Casp8:** caspase 8; **CCL2:** chemoattractant protein 1; **CDDP:** cisdiamminedichloroplatinum II; **CDK2:** cyclin-dependent kinase 2; **CKI:** cyclin-dependent kinases inhibitor; **CXCL6:** granulocyte chemotactic protein 2; **DNMT:** DNA methyltransferase; **EC:** epicatechin; **ECG:** epicatechingallate; **EFNA1:** ephrin A1; **EGC:** epigallocatechin; **EGCG:** epigallocatechingallate; **EGF:** epidermal growth factor; **EGFR:** epidermal growth factor receptor; **ER:** estrogen receptor; **ERK:** extracellular signal-regulated kinase; **ER-α:** estrogen receptor-α; **HDAC:** histone deacetylase; **HIF-1:** hypoxia-inducible factor 1; **HIV:** human immunodeficiency virus; **HPV:** human papillomavirus; **ID1:** inhibitor of DNA binding 1; **IFN-β1:** interferon β 1; **IGF-1:** insulin-like growth factor 1; **IGF-1R:** insulin-like growth factor receptor; **LMP:** lysosomal membrane permeabilization; **MMPs:** matrix metalloproteinases; **mTOR:** mammalian target of rapamycin; **PBD:** polo-box domain; **PDGFA:** platelet-derived growth factor α; **PI3K:** phosphoinositide-3-kinase; **PLK1:** Polo-like kinase 1; **RNA pol III:** RNA polymerase III; **ROS:** reactive oxygen species; **TGF-β2:** transforming growth factor β 2; **THBS-1:** thrombospondin 1; **TIMP-1:** tissue inhibitor of metalloproteinase-1; **TNFAIP2:** tumor necrosis factor α-induced protein 2; **Trx:** thioredoxin; **TrxR:** thioredoxin reductase; **VEGF:** vascular endothelial growth factor; **67LR:** 67KD laminin receptor.

## References

[1] World Health Organization Globocan2012: Estimated Cancer Incidence, Mortality and Prevalence worldwide in 2012. http://globocan.iarc.fr/Pages/fact_sheets_cancer.aspx.

[2] Waggoner S.E. (2003). Cervical cancer. Lancet.

[3] Moody C.A., Laimins L.A. (2010). Human papillomavirus oncoproteins: Pathways to transformation. Nat. Rev. Cancer.

[4] De Sanjose S., Quint W.G.V., Alemany L., Geraets D.T., Klaustermeier J.E., Lloveras B., Tous S., Felix A., Bravo L.E., Shin H.R. (2010). Human papillomavirus genotype attribution in invasive cervical cancer: A retrospective cross-sectional worldwide study. Lancet Oncol..

[5] Doorbar J., Quint W., Banks L., Bravo I.G., Stoler M., Broker T.R., Stanley M.A. (2012). The biology and life-cycle of human papillomaviruses. Vaccine.

[6] Bernard H.U., Burk R.D., Chen Z.G., Van Doorslaer K., Zur Hausen H., Ze Villiers E.M. (2010). Classification of papillomaviruses (pvs) based on 189 pv types and proposal of taxonomic amendments. Virology.

[7] Hirchaud F., Hermetet F., Ablise M., Fauconnet S., Vuitton D.A., Pretet J.L., Mougin C. (2013). Isoliquiritigenin induces caspase-dependent apoptosis via downregulation of hpv16 e6 expression in cervical cancer caski cells. Planta Med..

[8] Bosch F.X., Burchell A.N., Schiffman M., Giuliano A.R., De Sanjose S., Bruni L., Tortolero-Luna G., Kjaer S.K., Munoz N. (2008). Epidemiology and natural history of human papillomavirus infections and type-specific implications in cervical neoplasia. Vaccine.

[9] Melsheimer P., Vinokurova S., Wentzensen N., Bastert G., Doeberitz M.V. (2004). DNA aneuploidy and integration of human papillomavirus type 16 e6/e7 oncogenes in intraepithelial neoplasia and invasive squamous cell carcinoma of the cervix uteri. Clin. Cancer Res..

[10] Ziegert C., Wentzensen N., Vinokurova S., Kisseljov F., Einenkel J., Hoeckel M., Doeberitz M.V. (2003). A comprehensive analysis of hpv integration loci in anogenital lesions combining transcript and genome-based amplification techniques. Oncogene.

[11] Wilting S.M., Steenbergen R.D.M., Tijssen M., Van Wieringen W.N., Helmerhorst T.J.M., Van Kemenade F.J., Bleeker M.C.G., Van de Wiel M.A., Carvalho B., Meijer G.A. (2009). Chromosomal signatures of a subset of high-grade premalignant cervical lesions closely resemble invasive carcinomas. Cancer Res..

[12] Poljak M. (2012). Prophylactic human papillomavirus vaccination and primary prevention of cervical cancer: Issues and challenges. Clin. Microbiol. Infect..

[13] Alhamlan F.S., Al-Zahrani A.S., Almatrrouk S.A., Al-Ahdal M.N. (2017). Human papillomaviruses: The cervical cancer saga in developing countries. J. Infect. Dev. Ctries..

[14] Yuan C.H., Filippova M., Tungteakkhun S.S., Duerksen-Hughes P.J., Krstenansky J.L. (2012). Small molecule inhibitors of the hpv16-e6 interaction with caspase 8. Bioorg. Med. Chem. Lett..

[15] Vici P., Pizzuti L., Mariani L., Zampa G., Santini D., Di Lauro L., Gamucci T., Natoli C., Marchetti P., Barba M. (2016). Targeting immune response with therapeutic vaccines in premalignant lesions and cervical cancer: Hope or reality from clinical studies. Expert Rev. Vaccines.

[16] Liang Y.R., Ye Q., Jin J., Liang H., Lu J.L., Du Y.Y., Dong J.J. (2008). Chemical and instrumental assessment of green tea sensory preference. Int. J. Food Prop..

[17] Dong J.J., Ye J.H., Lu J.L., Zheng X.Q., Liang Y.R. (2011). Isolation of antioxidant catechins from green tea and its decaffeination. Food Bioprod. Process..

[18] Xiang L.P., Wang A., Ye J.H., Zheng X.Q., Polito C.A., Lu J.L., Li Q.S., Liang Y.R. (2016). Suppressive effects of tea catechins on breast cancer. Nutrients.

[19] Hou I.C., Amarnani S., Chong M.T., Bishayee A. (2013). Green tea and the risk of gastric cancer: Epidemiological evidence. World J. Gastroenterol..

[20] Arts I.C. (2008). A review of the epidemiological evidence on tea, flavonoids, and lung cancer. J. Nutr..

[21] Adhami V.M., Malik A., Zaman N., Sarfaraz S., Siddiqui I.A., Syed D.N., Afaq F., Pasha F.S., Saleem M., Mukhtar H. (2007). Combined inhibitory effects of green tea polyphenols and selective cyclooxygenase-2 inhibitors on the growth of human prostate cancer cells both in vitro and in vivo. Clin. Cancer Res..

[22] Lee S.C., Chan W.K., Lee T.W., Lam W.H., Wang X., Chan T.H., Wong Y.C. (2008). Effect of a prodrug of the green tea polyphenol (-)-epigallocatechin-3-gallate on the growth of androgen-independent prostate cancer in vivo. Nutr. Cancer.

[23] Chen X., Li Y., Lin Q., Wang Y., Sun H., Wang J., Cui G., Cai L., Dong X. (2014). Tea polyphenols induced apoptosis of breast cancer cells by suppressing the expression of survivin. Sci. Rep..

[24] Ni C.X., Gong H., Liu Y., Qi Y., Jiang C.L., Zhang J.P. (2017). Green tea consumption and the risk of liver cancer: A meta-analysis. Nutr. Cancer.

[25] Connors S.K., Chornokur G., Kumar N.B. (2012). New insights into the mechanisms of green tea catechins in the chemoprevention of prostate cancer. Nutr. Cancer.

[26] Pandey M., Shukla S., Gupta S. (2010). Promoter demethylation and chromatin remodeling by green tea polyphenols leads to re-expression of GSTP1 in human prostate cancer cells. Int. J. Cancer.

[27] Garcia F.A.R., Cornelison T., Nuno T., Greenspan D.L., Byron J.W., Hsu C.H., Alberts D.S., Chow H.H.S. (2014). Results of a phase II randomized, double-blind, placebo-controlled trial of polyphenon E in women with persistent high-risk hpv infection and low-grade cervical intraepithelial neoplasia. Gynecol. Oncol..

[28] Small W., Bacon M.A., Bajaj A., Chuang L.T., Fisher B.J., Harkenrider M.M., Jhingran A., Kitchener H.C., Mileshkin L.R., Viswanathan A.N. (2017). Cervical cancer: A global health crisis. Cancer.

[29] Zou C., Liu H., Feugang J.M., Hao Z., Chow H.H., Garcia F. (2010). Green tea compound in chemoprevention of cervical cancer. Int. J. Gynecol. Cancer.

[30] Singh M., Singh R., Bhui K., Tyagi S., Mahmood Z., Shukla Y. (2011). Tea polyphenols induce apoptosis through mitochondrial pathway and by inhibiting nuclear factor-kappaB and Akt activation in human cervical cancer cells. Oncol. Res..

[31] Ahn W.S., Huh S.W., Bae S.M., Lee I.P., Lee J.M., Namkoong S.E., Kim C.K., Sin J.I. (2003). A major constituent of green tea, EGCG, inhibits the growth of a human cervical cancer cell line, caski cells, through apoptosis, G1 arrest, and regulation of gene expression. DNA Cell Biol..

[32] Zhang L.F., Wu J.H., Ling M.T., Zhao L., Zhao K.N. (2015). The role of the PI3K/Akt/mTOR signalling pathway in human cancers induced by infection with human papillomaviruses. Mol. Cancer.

[33] Sah J.F., Balasubramanian S., Eckert R.L., Rorke E.A. (2004). Epigallocatechin-3-gallate inhibits epidermal growth factor receptor signaling pathway. Evidence for direct inhibition of ERK1/2 and Akt kinases. J. Biol. Chem..

[34] Pollak M.N., Schernhammer E.S., Hankinson S.E. (2004). Insulin-like growth factors and neoplasia. Nat. rev. Cancer.

[35] Li M., He Z., Ermakova S., Zheng D., Tang F., Cho Y.Y., Zhu F., Ma W.Y., Sham Y., Rogozin E.A. (2007). Direct inhibition of insulin-like growth factor-I receptor kinase activity by (-)-epigallocatechin-3-gallate regulates cell transformation. Cancer Epidemiol. Biomark. Prev..

[36] Chakrabarty S., Ganguli A., Das A., Nag D., Chakrabarti G. (2015). Epigallocatechin-3-gallate shows anti-proliferative activity in hela cells targeting tubulin-microtubule equilibrium. Chem. Biol. Interact..

[37] Liu X.Q., Erikson R.L. (2003). Polo-like kinase 1 in the life and death of cancer cells. Cell Cycle.

[38] Goh K.C., Wang H.S., Yu N.F., Zhou Y.F., Zheng Y., Lim Z.Y., Sangthongpitag K., Fang L.J., Du M., Wang X.K. (2004). PLK1 as a potential drug target in cancer therapy. Drug Dev. Res..

[39] Shan H.M., Shi Y., Quan J. (2015). Identification of green tea catechins as potent inhibitors of the polo-box domain of polo-like kinase 1. ChemMedChem.

[40] Blackburn E.H. (1992). Telomerases. Annu. Rev. Biochem..

[41] Shay J.W. (2016). Role of telomeres and telomerase in aging and cancer. Cancer Discov..

[42] Yokoyama M., Noguchi M., Nakao Y., Pater A., Iwasaka T. (2004). The tea polyphenol, (-)-epigallocatechin gallate effects on growth, apoptosis, and telomerase activity in cervical cell lines. Gynecol. Oncol..

[43] Noguchi M., Yokoyama M., Watanabe S., Uchiyama M., Nakao Y., Hara K., Iwasaka T. (2006). Inhibitory effect of the tea polyphenol, (-)-epigallocatechin gallate, on growth of cervical adenocarcinoma cell lines. Cancer Lett..

[44] Schramm L., Hernandez N. (2002). Recruitment of RNA polymerase III to its target promoters. Gene Dev..

[45] White R.J. (2004). RNA polymerase III transcription and cancer. Oncogene.

[46] Mital R., Kobayashi R., Hernandez N. (1996). RNA polymerase III transcription from the human U6 and adenovirus type 2 VAI promoters has different requirements for human BRF, a subunit of human TFIIIB. Mol. Cell. Biol..

[47] Teichmann M., Wang Z.X., Roeder R.G. (2000). A stable complex of a novel transcription factor IIB-related factor, human TFIIIB50, and associated proteins mediate selective transcription by RNA polymerase III of genes with upstream promoter elements. Proc. Natl. Acad. Sci. USA.

[48] Jacob J., Cabarcas S., Veras I., Zaveri N., Schramm L. (2007). The green tea component EGCG inhibits RNA polymerase III transcription. Biochem. Biophys. Res. Commun..

[49] Yokoyama M., Noguchi M., Nakao Y., Ysunaga M., Yamasaki F., Iwasaka T. (2008). Antiproliferative effects of the major tea polyphenol, (-)-epigallocatechin gallate and retinoic acid in cervical adenocarcinoma. Gynecol. Oncol..

[50] Kavallaris M. (2010). Microtubules and resistance to tubulin-binding agents. Nat. Rev. Cancer.

[51] Chakrabarty S., Das A., Bhattacharya A., Chakrabarti G. (2011). Theaflavins depolymerize microtubule network through tubulin binding and cause apoptosis of cervical carcinoma hela cells. J. Agric. Food Chem..

[52] Ji H., Liu N., Yin Y., Wang X., Chen X., Li J., Li J. (2018). Oxytocin inhibits ovarian cancer metastasis by repressing the expression of MMP-2 and VEGF. J. Cancer.

[53] Luo H.Q., Xu M., Zhong W.T., Cui Z.Y., Liu F.M., Zhou K.Y., Li X.Y. (2014). EGCG decreases the expression of HIF-1α and VEGF and cell growth in MCF-7 breast cancer cells. J. Buon.

[54] Li X.Y., Feng Y., Liu J.H., Feng X.W., Zhou K.Y., Tang X.D. (2013). Epigallocatechin-3-gallate inhibits IGF-I-stimulated lung cancer angiogenesis through downregulation of HIF-l α and VEGF expression. J. Nutrigenet. Nutr..

[55] Ferrara N., Gerber H.P., LeCouter J. (2003). The biology of VEGF and its receptors. Nat. Med..

[56] De Francesco E.M., Sims A.H., Maggiolini M., Sotgia F., Lisanti M.P., Clarke R.B. (2017). GPER mediates the angiocrine actions induced by IGF1 through the HIF-1 α/VEGF pathway in the breast tumor microenvironment. Breast Cancer Res..

[57] Wang G.L., Jiang B.H., Rue E.A., Semenza G.L. (1995). Hypoxia-inducible factor 1 is a basic-helix-loop-helix-PAS heterodimer regulated by cellular O2 tension. Proc. Natl. Acad. Sci. USA.

[58] Carmeliet P., Dor Y., Herbert J.M., Fukumura D., Brusselmans K., Dewerchin M., Neeman M., Bono F., Abramovitch R., Maxwell P. (1998). Role of HIF-1α in hypoxia-mediated apoptosis, cell proliferation and tumour angiogenesis. Nature.

[59] Krishnamachary B., Berg-Dixon S., Kelly B., Agani F., Feldser D., Ferreira G., Iyer N., LaRusch J., Pak B., Taghavi P. (2003). Regulation of colon carcinoma cell invasion by hypoxia-inducible factor 1. Cancer Res..

[60] Frezza M., Schmitt S., Dou Q.P. (2011). Targeting the ubiquitin-proteasome pathway: An emerging concept in cancer therapy. Curr. Top. Med. Chem..

[61] Zhang Q., Tang X., Lu Q., Zhang Z., Rao J., Le A.D. (2006). Green tea extract and (-)-epigallocatechin-3-gallate inhibit hypoxia- and serum-induced HIF-1α protein accumulation and VEGF expression in human cervical carcinoma and hepatoma cells. Mol. Cancer Ther..

[62] Joyce J.A. (2005). Therapeutic targeting of the tumor microenvironment. Cancer Cell.

[63] Park C.C., Bissell M.J., Barcellos-Hoff M.H. (2000). The influence of the microenvironment on the malignant phenotype. Mol. Med. Today.

[64] Wilson K.E., Li Z.Q., Kara M., Gardner K.L., Roberts D.D. (1999). β(1) integrin- and proteoglycan-mediated stimulation of T lymphoma cell adhesion and mitogen-activated protein kinase signaling by thrombospondin-1 and thrombospondin-1 peptides. J. Immunol..

[65] Kleine-Lowinski K., Gillitzer R., Kuhne-Heid R., Rosl F. (1999). Monocyte-chemo-attractant-protein-1 (MCP-1)-gene expression in cervical intra-epithelial neoplasias and cervical carcinomas. Int. J. Cancer.

[66] Cui X.D., Lee M.J., Yu G.R., Kim I.H., Yu H.C., Song E.Y., Kim D.G. (2010). EFNA1 ligand and its receptor EphA2: Potential biomarkers for hepatocellular carcinoma. Int. J. Cancer.

[67] Le Jan S., Amy C., Cazes A., Monnot C., Lamande N., Favier J., Philippe J., Sibony M., Gasc J.M., Corvol P. (2003). Angiopoietin-like 4 is a proangiogenic factor produced during ischemia and in conventional renal cell carcinoma. Am. J. Pathol..

[68] Kaido T., Bandu M.T., Maury C., Ferrantini M., Belardelli F., Gresser I. (1995). IFN-α(1) gene transfection completely abolishes the tumorigenicity of murine B16 melanoma-cells in allogeneic DBA/2 mice and decreases their tumorigenicity in syngeneic C57BL/6 mice. Int. J. Cancer.

[69] Sidky Y.A., Borden E.C. (1987). Inhibition of angiogenesis by interferons-effects on tumor-induced and lymphocyte-induced vascular-responses. Cancer Res..

[70] Jovanovic M., Stefanoska I., Radojcic L., Vicovac L. (2010). Interleukin-8 (CXCL8) stimulates trophoblast cell migration and invasion by increasing levels of matrix metalloproteinase (MMP)2 and MMP9 and integrins α(5) and β(1). Reproduction.

[71] Tudoran O., Soritau O., Balacescu O., Balacescu L., Braicu C., Rus M., Gherman C., Virag P., Irimie F., Berindan-Neagoe I. (2012). Early transcriptional pattern of angiogenesis induced by EGCG treatment in cervical tumour cells. J. Cell. Mol. Med..

[72] Gonzalez S.L., Stremlau M., He X., Basile J.R., Munger K. (2001). Degradation of the retinoblastoma tumor suppressor by the human papillomavirus type 16 E7 oncoprotein is important for functional inactivation and is separable from proteasomal degradation of E7. J. Virol..

[73] Yokoyama M., Tsutsumi K., Pater A., Pater M.M. (1994). Human papillomavirus 18-immortalized endocervical cells with in-vitro cytokeratin expression characteristics of adenocarcinoma. Obstet. Gynecol..

[74] Kuhn D.J., Burns A.C., Kazi A., Dou Q.P. (2004). Direct inhibition of the ubiquitin-proteasome pathway by ester bond-containing green tea polyphenols is associated with increased expression of sterol regulatory element-binding protein 2 and LDL receptor. Biochim. Biophys. Acta.

[75] Wang J., Sampath A., Raychaudhuri P., Bagchi S. (2001). Both Rb and E7 are regulated by the ubiquitin proteasome pathway in HPV-containing cervical tumor cells. Oncogene.

[76] Bonfili L., Cuccioloni M., Mozzicafreddo M., Cecarini V., Angeletti M., Eleuteri A.M. (2011). Identification of an EGCG oxidation derivative with proteasome modulatory activity. Biochimie.

[77] Qiao Y.Y., Cao J.Y., Xie L.Q., Shi X.L. (2009). Cell growth inhibition and gene expression regulation by (-)-epigallocatechin-3-gallate in human cervical cancer cells. Arch. Pharm. Res..

[78] Giannelli G., Antonaci S. (2005). MMP and TIMP assay in cancer: Biological and clinical significance. Int. J. Cancer.

[79] GomisRuth F.X., Maskos K., Betz M., Bergner A., Huber R., Suzuki K., Yoshida N., Nagase H., Brew K., Bourenkov G.P. (1997). Mechanism of inhibition of the human matrix metalloproteinase stromelysin-1 by TIMP-1. Nature.

[80] Sharma C., Nusri Q.E.A., Begum S., Javed E., Rizvi T.A., Hussain A. (2012). (-)-Epigallocatechin-3-gallate induces apoptosis and inhibits invasion and migration of human cervical cancer cells. Asian Pac. J. Cancer.

[81] Roomi M.W., Monterrey J.C., Kalinovsky T., Rath M., Niedzwiecki A. (2010). In vitro modulation of MMP-2 and MMP-9 in human cervical and ovarian cancer cell lines by cytokines, inducers and inhibitors. Oncol. Rep..

[82] Siddiqui F.A., Naim M., Islam N. (2011). Apoptotic effect of green tea polyphenol (EGCG) on cervical carcinoma cells. Diagn. Cytopathol..

[83] Al-Hazzani A.A., Alshatwi A.A. (2011). Catechin hydrate inhibits proliferation and mediates apoptosis of siha human cervical cancer cells. Food Chem. Toxicol..

[84] Finkel T. (2003). Oxidant signals and oxidative stress. Curr. Opin. Cell Biol..

[85] Beckman K.B., Ames B.N. (1997). Oxidative decay of DNA. J. Biol. Chem..

[86] Azam S., Hadi N., Khan N.U., Hadi S.M. (2004). Prooxidant property of green tea polyphenols epicatechin and epigallocatechin-3-gallate: Implications for anticancer properties. Toxicol. In Vitro.

[87] Li G.X., Chen Y.K., Hou Z., Xiao H., Jin H.Y., Lu G., Lee M.J., Liu B., Guan F., Yang Z.H. (2010). Pro-oxidative activities and dose-response relationship of (-)-epigallocatechin-3-gallate in the inhibition of lung cancer cell growth: A comparative study in vivo and in vitro. Carcinogenesis.

[88] Krstic M., Stojadinovic M., Smiljanic K., Stanic-Vucinic D., Velickovic T.C. (2015). The anti-cancer activity of green tea, coffee and cocoa extracts on human cervical adenocarcinoma hela cells depends on both pro-oxidant and anti-proliferative activities of polyphenols. RSC Adv..

[89] Zhang H., Cao D., Cui W., Ji M., Qian X., Zhong L. (2010). Molecular bases of thioredoxin and thioredoxin reductase-mediated prooxidant actions of (-)-epigallocatechin-3-gallate. Free Radic. Biol. Med..

[90] Zorov D.B., Juhaszova M., Sollott S.J. (2014). Mitochondrial reactive oxygen species (ROS) and ROS-induced ROS release. Physiol. Rev..

[91] Chung Y.M., Bae Y.S., Lee S.Y. (2003). Molecular ordering of ROS production, mitochondrial changes, and caspase activation during sodium salicylate-induced apoptosis. Free Radic. Biol. Med..

[92] Singh M., Tyagi S., Bhui K., Prasad S., Shukla Y. (2010). Regulation of cell growth through cell cycle arrest and apoptosis in HPV 16 positive human cervical cancer cells by tea polyphenols. Investig. New Drug..

[93] Luzio J.P., Pryor P.R., Bright N.A. (2007). Lysosomes: Fusion and function. Nat. Rev. Cell Biol..

[94] Zhang Y., Yang N.D., Zhou F., Shen T., Duan T., Zhou J., Shi Y., Zhu X.Q., Shen H.M. (2012). (-)-Epigallocatechin-3-gallate induces non-apoptotic cell death in human cancer cells via ROS-mediated lysosomal membrane permeabilization. PLoS ONE.

[95] Ho Y.P., Au-Yeung S.C.F., To K.K.W. (2003). Platinum-based anticancer agents: Innovative design strategies and biological perspectives. Med. Res. Rev..

[96] Arany I., Safirstein R.L. (2003). Cisplatin nephrotoxicity. Semin. Nephrol..

[97] Matsushima H., Yonemura K., Ohishi K., Hishida A. (1998). The role of oxygen free radicals in cisplatin-induced acute renal failure in rats. J. Lab. Clin. Med..

[98] Kilic U., Sahin K., Tuzcu M., Basak N., Orhan C., Elibol-Can B., Kilic E., Sahin F., Kucuk O. (2014). Enhancement of cisplatin sensitivity in human cervical cancer: Epigallocatechin-3-gallate. Front. Nutr..

[99] Singh M., Bhui K., Singh R., Shukla Y. (2013). Tea polyphenols enhance cisplatin chemosensitivity in cervical cancer cells via induction of apoptosis. Life Sci..

[100] Singh M., Bhatnagar P., Srivastava A.K., Kumar P., Shukla Y., Gupta K.C. (2011). Enhancement of cancer chemosensitization potential of cisplatin by tea polyphenols poly(lactide-co-glycolide) nanoparticles. J. Biomed. Nanotechnol..

[101] Alshatwi A.A., Athinarayanan J., Subbarayan P.V. (2015). Green synthesis of platinum nanoparticles that induce cell death and G2/m-phase cell cycle arrest in human cervical cancer cells. J. Mater. Sci. Mater. Med..

[102] Burger R.M., Peisach J., Horwitz S.B. (1981). Activated bleomycin-a transient complex of drug, iron, and oxygen that degrades DNA. J. Biol. Chem..

[103] Sleijfer S. (2001). Bleomycin-induced pneumonitis. Chest.

[104] Hay J., Shahzeidi S., Laurent G. (1991). Mechanisms of bleomycin-induced lung damage. Arch. Toxicol..

[105] Vorechovsky I., Munzarova M., Lokaj J. (1989). Increased bleomycin-induced chromosome-damage in lymphocytes of patients with common variable immunodeficiency indicates an involvement of chromosomal instability in their cancer predisposition. Cancer Immunol. Immunothera..

[106] Alshatwi A.A., Periasamy V.S., Athinarayanan J., Elango R. (2016). Synergistic anticancer activity of dietary tea polyphenols and bleomycin hydrochloride in human cervical cancer cell: Caspase-dependent and independent apoptotic pathways. Chem. Biol. Interact..

[107] Tallen G., Riabowol K. (2014). Keep-ING balance: Tumor suppression by epigenetic regulation. FEBS Lett..

[108] Khan M.A., Hussain A., Sundaram M.K., Alalami U., Gunasekera D., Ramesh L., Hamza A., Quraishi U. (2015). (-)-Epigallocatechin-3-gallate reverses the expression of various tumor-suppressor genes by inhibiting DNA methyltransferases and histone deacetylases in human cervical cancer cells. Oncol. Rep..

[109] Virksaite A., Bakutyte S., Navakauskiene R. (2016). Assessment of apoptosis and senescence in acute myeloid leukemia NB-4 cells treated with epigenetic modifiers EGCG and BIX-01294. Eur. J Cancer.

[110] Subramaniam D., Thombre R., Dhar A., Anant S. (2014). DNA methyltransferases: A novel target for prevention and therapy. Front. Oncol..

[111] Sobel M.E. (1993). Differential expression of the 67 kDa laminin receptor in cancer. Semin. Cancer Biol..

[112] Kumazoe M., Sugihara K., Tsukamoto S., Huang Y.H., Tsurudome Y., Suzuki T., Suemasu Y., Ueda N., Yamashita S., Kim Y. (2013). 67-kDa laminin receptor increases cGMP to induce cancer-selective apoptosis. J. Clin. Investig..

[113] Gundimeda U., McNeill T.H., Fan T.K., Deng R., Rayudu D., Chen Z., Cadenas E., Gopalakrishna R. (2014). Green tea catechins potentiate the neuritogenic action of brain-derived neurotrophic factor: Role of 67-kDa laminin receptor and hydrogen peroxide. Biochem. Biophys. Res. Commun..

[114] Umeda D., Tachibana H., Yamada K. (2005). Epigallocatechin-3-*o*-gallate disrupts stress fibers and the contractile ring by reducing myosin regulatory light chain phosphorylation mediated through the target molecule 67 kDa laminin receptor. Biochem. Biophys. Res. Commun..

[115] Umeda D., Yano S., Yamada K., Tachibana H. (2008). Green tea polyphenol epigallocatechin-3-gallate signaling pathway through 67-kDa laminin receptor. J. Biol. Chem..

[116] Tsukamoto S., Yamashita S., Kim Y.H., Kumazoe M., Huang Y., Yamada K., Tachibana H. (2012). Oxygen partial pressure modulates 67-kDa laminin receptor expression, leading to altered activity of the green tea polyphenol, EGCG. FEBS Lett..

[117] Moradzadeh M., Hosseini A., Erfanian S., Rezaei H. (2017). Epigallocatechin-3-gallate promotes apoptosis in human breast cancer T47D cells through down-regulation of PI3K/AKT and telomerase. Pharmacol. Rep..

[118] Liu L., Zuo J., Wang G.D. (2017). Epigallocatechin-3-gallate suppresses cell proliferation and promotes apoptosis in Ec9706 and Eca109 esophageal carcinoma cells. Oncol. Lett..

[119] Wang J., Liu L., Ma H.W. (2017). Label-free real-time investigation of the effect of telomerase inhibitors based on quartz crystal microbalance measurement. Sens. Actuators B Chem..

[120] Stern J.L., Theodorescu D., Vogelstein B., Papadopoulos N., Cech T.R. (2015). Mutation of the TERT promoter, switch to active chromatin, and monoallelic TERT expression in multiple cancers. Genes Dev..

[121] Circu M., Cardelli J., Barr M.P., O’Byrne K., Mills G., El-Osta H. (2018). Modulating lysosomal function through lysosome membrane permeabilization or autophagy suppression restores sensitivity to cisplatin in refractory non-small-cell lung cancer cells (vol 12, e0184922, 2017). PLoS ONE.

[122] Kaminskas E., Farrell A.T., Wang Y.C., Sridhara R., Pazdur R. (2005). FDA drug approval summary: Azacitidine (5-azacytidine, vidaza^TM^) for injectable suspension. Oncologist.

[123] Jia Y., Hu T., Hang C.Y., Yang R., Li X., Chen Z.L., Mei Y.D., Zhang Q.H., Huang K.C., Xiang Q.Y. (2012). Case-control study of diet in patients with cervical cancer or precancerosis in Wufeng, a high incidence region in China. Asian Pac. J. Cancer Prev..

